# On the Relation between Prediction and Imputation Accuracy under Missing Covariates

**DOI:** 10.3390/e24030386

**Published:** 2022-03-09

**Authors:** Burim Ramosaj, Justus Tulowietzki, Markus Pauly

**Affiliations:** Faculty of Statistics, TU Dortmund University, Joseph-Von-Fraunhofer Str. 2-4, 44227 Dortmund, Germany; justus.tulowietzki@tu-dortmund.de (J.T.); markus.pauly@tu-dortmund.de (M.P.)

**Keywords:** missing covariates, imputation accuracy, prediction accuracy, prediction intervals, bagging, boosting

## Abstract

Missing covariates in regression or classification problems can prohibit the direct use of advanced tools for further analysis. Recent research has realized an increasing trend towards the use of modern Machine-Learning algorithms for imputation. This originates from their capability of showing favorable prediction accuracy in different learning problems. In this work, we analyze through simulation the interaction between imputation accuracy and prediction accuracy in regression learning problems with missing covariates when Machine-Learning-based methods for both imputation and prediction are used. We see that even a slight decrease in imputation accuracy can seriously affect the prediction accuracy. In addition, we explore imputation performance when using statistical inference procedures in prediction settings, such as the coverage rates of (valid) prediction intervals. Our analysis is based on empirical datasets provided by the UCI Machine Learning repository and an extensive simulation study.

## 1. Introduction

The presence of missing values in data preparation and data analysis makes the use of state-of-the art statistical methods difficult to apply. Seeking a universal answer to such problems was the main idea of [1], who introduced (multiple) imputation. Through imputation, one provides data analysts (sequences) of completed datasets, based on which, various data analysis procedures can be conducted. An alternative to imputation is the use of so-called data adjustment methods: statistical methods that directly treat missing instances during training or parameter estimation, such as the full-information-maximum-likelihood method (see, e.g., [2]) or the expectation-maximization algorithm (cf. [3]).

A large disadvantage of these methods is the expertise knowledge on theoretical model construction, where the likelihood function of parameters of interest needs to be adopted appropriately in order to account for missing information. Such examples can be found in [4,5,6], where whole statistical testing procedures were adjusted to account for missing values. It is already well-known that more naive methods, such as list-wise deletion or mean imputation can lead to severe estimation bias, see, e.g., [1,7,8,9,10]. Therefore, we do not discuss these approaches further.

In the current paper, we focus on regression problems, where we do not have complete information on the set of covariates. Missing covariates in supervised regression learning have been part in a variety of theoretical and applicative research fields. In [10], for example, a theoretical analysis based on maximum semiparametric likelihood for constructing consistent regression estimates was conducted. While in [11,12] or [13], for example, multiple imputation is used as a tool in medical research for variable selection or bias-reduction in parameter estimation. More recent research has focused on Machine-Learning (ML)-based imputation.

In [14,15,16,17], for example, the Random Forest method was used to impute missing values in various datasets with mixed variable scales under the assumption of independent measurements. Other ML-based methods, such as the *k*-nearest neighbor method, boosting machines and Bayesian Regression in combination with classification and regression trees, have been part of (multiple) imputation, see, e.g., [18,19,20,21,22].

Modeling dependencies in imputation methods for multi-block time series data or repeated measurement designs is, however, a non-trivial underpinning. Imputation methods for such time series data can be found in, e.g., [23,24] or [25], for example. Therein, a special focus has been placed on (data) matrix factorization methods, such as the singular value decomposition. In our setting, however, a focus is placed on matrix completion methods using ML-approaches, such as tree-based algorithms.

Choosing an appropriate imputation method for missing data problems usually depends on several aspects, such as the capability to treat mixed-type data, to impute reasonable values in variables of, e.g., bounded support, and to provide a fast imputation solution. Imputation accuracy can usually be assessed through the consideration of performance measures. Here, depending on the subsequent application, one may focus on the data reproducibility measured through the normalized root mean squared error (NRMSE) and proportion of false classification (PFC) or distribution preserving measures, such as Kolmogorov–Smirnov based statistics, see, e.g., [14,21,26].

It is important to realize that these two classes of performance measures for the evaluation of imputation do not always agree, see, e.g., [26,27]. In fact, one could be provided by an imputation scheme with comparably low NRMSE values, for which subsequent statistical inference can have highly-inflated type-I error rates. Therefore, choosing imputation methods by solely focusing on data reproducibility will not always lead to correct inference procedures. To control for the latter, [1] coined the term *proper imputation*: a property that guarantees correct statistical inference under the (multiple) imputation scheme.

While imputation accuracy can be accessed through data reproducibility or distributional recovery, the prediction performance of subsequently applied ML methods (i.e., after imputation) is often evaluated using the mean-squared error (MSE) or the misclassification error (MCE). Under missing covariates, however, the sole focus on these measures is not sufficient, as the disagreement between data reproducibility and statistical inference has shown. In fact, beyond point-prediction, we may also be interested in uncertainty quantifiation in the form of prediction intervals. The effect of missing covariates on the latter remains mostly unknown. In this paper, we aim to close this gap in empirical and simulation-based scenarios.

We thereby place a special focus on the relation between data reproducibility and correct statistical inference for post-imputation prediction. While the latter could also be measured by distributional discrepancies as in [26], we aim to place a special focus on coverage rates and prediction interval lengths in post-imputation prediction instead. The reason for this is the rise in ML-based imputation methods and their competitive predictive performance for both imputation and prediction. Taking into account that data reproducibility and statistical inference are not always in harmony under the missing framework, an interesting question remains whether appropriate data reproducible imputation schemes lead to favourable prediction results after imputation.

Furthermore, it is unknown whether imputation schemes with comparably low NRMSE values will lead to accurate predictive machines in terms of delivering appropriate point predictions for future outcomes while also correctly quantifying their uncertainty. Therefore, based on different ML methods in supervised regression learning problems, we

(i) aim to clarify the interaction between NRMSE and MSE as measures for data reproducibility and predictive post-imputation ability. We estimate both measures on various missing rates, imputation methods and prediction models to account for potential interactions between the NRMSE and MSE.
(ii) Furthermore, we aim to enlighten the general issue whether imputation methods with comparably low imputation errors also lead to correct predictive inference. That is, we analyze the impact of accurate data-reproducible imputation schemes on predictive, post-imputation statistical inference in terms of correct uncertainty quantification. To measure the latter, we take into account correct coverage rates and (narrow) interval lengths of point-wise prediction intervals in post-imputation settings obtained through ML methods.

## 2. Measuring Accuracy

Measuring imputation accuracy can happen in many ways. A great deal of research has been focused on the general idea of *reconstructing* missing instances and being as *close* as possible to the true underlying data (cf. [14,21]).

Although this approach seems reasonable, several disadvantages have been discovered when using data reproducible measures, such as squared error loss, especially for later statistical inference; see, e.g., [1,26,27]. Therefore, we discuss several suitable measures for assessing prediction accuracy in regression learning problems with missing covariates. In the sequel, we assume that we have access to iid data collected in $D_{n}=\left\{{\left[X_{i}^{⊤},Y_{i}\right]}^{⊤}\in R^{p+1}:i=1,\ldots ,n\right\}$, where

(1) $$\begin{array}{c} Y_{i}=m\left(X_{i}\right)+\epsilon_{i}. \end{array}$$

Here, $m\left(x\right)=E\left[Y_{1}|X_{1}=x\right]$ is the regression function, ${\left\{\epsilon_{i}\right\}}_{i=1}^{n}$ is a sequence of iid random variables with $E[\epsilon_{1}]=0$ and $Var\left(\epsilon_{1}\right)=\sigma^{2}\in \left(0,\infty \right)$ and we assume continuous covariates $X_{i}$. Missing positions in the features ${\left\{X_{i}\right\}}_{i=1}^{n}$ are modeled by the indicator matrix $R={\left\{R_{ij}\right\}}_{ij}\in {\left\{0,1\right\}}^{n\times p}$, where $R_{ij}=0$ indicates that the *i*-th observation of feature j∈{1,…,p} is not observable. Focusing on the general issue of predicting outcomes in regression learning for new feature outcomes, we restrict our attention to data reproducible accuracy measures and model prediction accuracy measures. In order to cover statistical inference correctness for prediction, we use ML-based prediction intervals as proposed in [28,29,30] to account for coverage rates and interval lengths.

### 2.1. Imputation and Prediction Accuracy

In our setting, (missing) covariates are continuously scaled leading to the use of accuracy measures for continuous random variables. Regarding imputation accuracy, we consider the NRMSE formally given by

(2) $$\begin{array}{cc} NRMSE & =\frac{\sqrt{\sum_{\left(i,j\right)\in N_{mis}}{\left(X_{ij}^{imp}-X_{ij}^{mis}\right)}^{2}}}{\sqrt{\sum_{\left(i,j\right)\in N_{mis}}{\left(X_{ij}^{imp}-{\bar{X}}_{\cdot \cdot }^{mis}\right)}^{2}}}, \end{array}$$

where $N_{mis}=\left\{\left(i,j\right)\in \left\{1,\ldots ,n\right\}\times \left\{1,\ldots ,p\right\}|R_{ij}=0\right\}$ is the set of all observations and features with missing entries in those positions. Here, $X_{ij}^{imp}$ denotes the imputed value of observation *i* for variable *j*, while $X_{ij}^{mis}$ is the true, unobserved component of those positions. $X_{\cdot \cdot }^{mis}$ is the mean of the sequence $\left\{X_{ij}:R_{ij}=0\right\}$. Regarding the overall model performance on prediction, we make use of the mean squared error

(3) $$\begin{array}{cc} MSE & =E[{\left(Y-{\hat{m}}_{n}\left(X\right)\right)}^{2}], \end{array}$$

where ${\hat{m}}_{n}$ is an ML-based estimator of *m* on $D_{n}$ and ${\left[X^{⊤},Y\right]}^{⊤}$ is an independent copy of ${\left[X_{1}^{⊤},Y_{1}\right]}^{⊤}$. Note that, in the missing framework, *m* is estimated on the imputed dataset $D_{n}^{imp}$, while the MSE is (usually) estimated using cross-validation procedures.

### 2.2. Prediction Intervals

Based on the methods for uncertainty quantification proposed in [28,29,30,31], we make use of Random-Forest-based prediction intervals. In an extensive simulation study in [30], it could be seen that other, ML-based prediction intervals, such as the (stochastic) gradient tree boosting (cf. [32]) or the XGBoost method (cf. [33]) did not perform well under completely observed covariates. Therefore, we restrict our attention to those already indicating accurate coverage rates in completely observed settings. Meinshausen’s Quantile Regression Forest (see [28]), for example, delivers a point-wise prediction interval for an unseen feature point X=x, which is formally given by

(4) $$\begin{array}{cc} PI_{QRF,n} & =[{\hat{Q}}_{n,\alpha /2}\left(x\right);\;{\hat{Q}}_{n,1-\alpha /2}\left(x\right)], \end{array}$$

where ${\hat{Q}}_{n,\alpha /2}\left(x\right)=\inf\left\{y|{\hat{F}}_{n}\left(y|x\right)\geq \alpha /2\right\}$ and ${\hat{F}}_{n}\left(y|x\right)$ is a Random-Forest-based estimator for the conditional distribution function F(y|x) of Y|X=x. Other prediction intervals based on the Random Forest are, e.g., given in [29,30]. Following the same notation as in [30], we refer with ${\hat{m}}_{n,M}\left(x\right)$ to a Random Forest prediction at x, trained on $D_{n}$ using *M* decision trees, while $z_{1-\alpha }$ is the corresponding quantile of the standard normal distribution. We consider the same residual variance estimators as in [30], where ${\hat{\sigma }}_{n,M}$ is the trivial residual variance estimate, ${\hat{\sigma }}_{n,Mcorrect}$ is the residual variance estimate with finite-*M* bias correction and ${\hat{\sigma }}_{n,M;W}$ is a weighted residual variance estimator, see also [31]. Moreover, we denote with ${\hat{D}}_{n,\alpha /2}^{RF}$ the empirical quantile of the Random Forest Out-of-Bag residuals. With this notation, we obtain four more prediction intervals:

(5) $$\begin{array}{cc} PI_{n,empQ}\left(x\right) & =[{\hat{m}}_{n,M}\left(x\right)+{\hat{D}}_{n,\alpha /2}^{RF};\;{\hat{m}}_{n,M}\left(x\right)+{\hat{D}}_{n,1-\alpha /2}^{RF}], \end{array}$$

(6) $$\begin{array}{cc} PI_{n,ResVar}\left(x\right) & =[{\hat{m}}_{n,M}\left(x\right)-z_{1-\alpha /2}\cdot {\hat{\sigma }}_{n,M};\;{\hat{m}}_{n,M}\left(x\right)+z_{1-\alpha /2}\cdot {\hat{\sigma }}_{n,M}], \end{array}$$

(7) $$\begin{array}{cc} PI_{n,Mcorrect}\left(x\right) & =[{\hat{m}}_{n,M}\left(x\right)-z_{1-\alpha /2}\cdot {\hat{\sigma }}_{n,Mcorrect};\;{\hat{m}}_{n,M}\left(x\right)+z_{1-\alpha /2}\cdot {\hat{\sigma }}_{n,Mcorrect}], \end{array}$$

(8) $$\begin{array}{cc} PI_{n,weighted}\left(x\right) & =[{\hat{m}}_{n,M}\left(x\right)-z_{1-\alpha /2}\cdot {\hat{\sigma }}_{n,M;W};\;{\hat{m}}_{n,M}\left(x\right)+z_{1-\alpha /2}\cdot {\hat{\sigma }}_{n,M;W}]. \end{array}$$

For benchmarking, we additionally consider a prediction interval obtained under the linear model assumption. Imputation accuracy in inferential prediction under missing covariates is then assessed by considering Monte-Carlo estimated coverage rates and interval lengths.

## 3. Imputation and Prediction Models

We made use of the following state-of-the-art ML regression models for prediction

- the Random Forest as implemented in the R-package ranger (c.f [17]),
- the (stochastic) gradient tree boosting (SGB) method from the R-package gbm (cf. [32]) and
- the XGBoost method, also known as Queen of ML (cf. [34]), as implemented in the R-package xgboost.

For each of them, we fit a prediction model to the (imputed) data. Both boosting methods rely on additive regression trees that are fitted sequentially using the principles of gradient descent for loss minimization. XGBoost, however, is slightly different by introducing extra randomization in tree construction, a proportional shrinkage on the leaf nodes and a clever penalization of trees. We refer to [32,33,35] for details on the concrete algorithms. For benchmarking, a linear model is trained as well.

Although several imputation models are available on various (statistical) software packages, we place a special focus on Random-Forest-based imputation schemes and the multivariate imputation using chained equations (MICE) procedure (cf. [11,14,21]). The reasons for this are twofold, but both have roots in the same theoretical issue called *congeniality*, see [36] for a formal definition. In its core, congeniality in (multiple) imputation refers to the existence of a Bayesian model such that

1. the posterior mean and posterior variance of the parameter of interest agrees with the point estimator resp. its variance estimator calculated under the analysis model and
2. the conditional distribution of the missing observations given the observed points under the considered Bayesian model agrees with the imputation model.

Therefore, congeniality builds a bridge between the imputation and analysis procedure by assuming the existence of a larger model that is compatible with both—the analysis and imputation model. If ML methods are used during the analysis phase, the compatibility in terms of congeniality is non-trivial. Using the same methods during the imputation and analysis phase, however, can ease the verification through the use of the same model during imputation and analysis.

Hence, a potential disagreement of imputation and prediction models, however, can result in uncongenial (multiple) imputation methods. The latter yields to invalid (multiple) imputation inference, as can be seen in [37] or [36], for example. Secondly, focusing on Bayesian models for imputation, such as the MICE procedure, is in line with the general framework of congeniality and the idea of (multiple) imputation. Although we do not directly compute point-estimates during the analysis phase, interesting quantities in our framework are Random-Forest-based prediction intervals and estimators of the MSE.

missForest in R is an iterative algorithm developed by [14] that imputes continuous and discrete random variables using trained Random Forests on complete subsets of the data and imputes missing values through prediction with the trained Random-Forest model. The process iterates in imputing missing values until a pre-defined stopping criterion is met. Similar to the missForest algorithm, we substituted the core learning method with other ML-based methods, such as the SGB method (in the sequel referred to as the gbm for the algorithmic implementation) and the XGBoost method (in the sequel referred to with xgboost for the algorithmic implementation).

Both methods are implemented in R using the same algorithmic framework as missForest, while substituting the Random Forest method with the SGB resp. XGBoost. That means that we train the SGB resp. XGBoost on (complete) subsets of the data and impute missing values through the prediction of the trained model in an iterative fashion.

MICE is a family of Bayesian imputation models developed in [38,39]. Under the normality assumption (i.e., MICE NORM), the method assumes a (Bayesian) linear regression model, where every parameter in that model is drawn from suitable priors. The predictive mean matching approach (MICE PMM) is similar to MICE NORM but does not impute missing values through the prediction of those points using the Bayesian linear model and instead randomly selects among observed points that are closest to the same model prediction as MICE NORM. In addition to these methods, MICE enables the implementation of Random-Forest-based methods, referred to as MICE RF, see, e.g., [40].

The latter assumes a modified Random Forest, where additional randomization is applied compared to the missForest. For example, instead of simply predicting missing values through averaging observations in leaf nodes, the method randomly selects them. In addition, in the complete subset of the data determined for training the Random Forest, potential missing values are not initially imputed by mean or mode values but by random draws among observed values. In the sequel, we refer to the algorithmic implementation in R of all these methods using the terms mice_norm, mice_pmm and mice_rf.

## 4. Simulation Design

Our simulation design is separated in two parts. In the first part, empirical data from the UCI Machine Learning Repository covering regression learning problems are considered for the purpose of measuring imputation and prediction accuracy. We focused on selecting datasets from the repository that reveal a high amount of continous variables, while reflecting both time series data and observations measured as independent and identical realizations of random variables with different dimensions. Summary statistics of every dataset can be found in Appendix A. The following five datasets are considered:

1. The **Airfoil Data** consists of (p+1)=6 variables measured in n=1503 observations, where the target variable is the scaled sound pressure level measured in decibels. The aim of this study conducted by NASA was to detect the impact of physical shapes of airfoils on the produced noise. The data consists of several blades measured in different experimental scenarios, such as various wind attack angles, free-stream velocities and frequencies. We may assume iid observations ${\left[X_{i}^{⊤},Y_{i}\right]}^{⊤}$, i=1,…,n for every experimental setting.
2. In the **Concrete Data**, (p+1)=7 variables are measured in n=1030 observations. The target variable is the concrete compressive strength measured in MPa units. Different mixing components, such as cement, water and fly ash, for example, are used to measure the concrete strength. It is reasonable to assume iid realizations from ${\left[X_{i}^{⊤},Y_{i}\right]}^{⊤}$ for i=1,…,n.
3. The aim in the **QSAR Data** is to predict aquatic toxicity for a certain fish species. It consists of (p+1)=9 variables measured in n=546 observations. The n=546 observations can be considered as iid realizations of ${\left[X_{i}^{⊤},Y_{i}\right]}^{⊤}$ for i=1,…,n, while all features and the response are continuous.
4. The **Real Estate Data** has (p+1)=7 variables and n=413 observations. The aim is to build a prediction model for house price developments in the area of New Taipei City in Taiwan. Different features, such as the house age or the location measured as a bivariate coordinate vector, for example, are measured for building a prediction model. In our simulation, we dropped the variable *transaction date* and assumed an row-wise iid structure. The dataset, however, can also be considered as time series data.
5. The **Power Plant Data** consists of n=9568 observations with (p+1)=5 variables. The actual dataset is much larger in terms of observations; however, only the first 9568 are selected to speed the computations. The aim of this dataset is to predict the electric power generation of a water power-plant in Turkey. This dataset is different from the previous ones due to its time series structure. The dataset can be considered as multiple time series measured in five different variables.

For each dataset, missing values under the MCAR scheme were inserted to the (n×p)-dimensional dataset with r∈{0.1,0.2,0.3,0.5,0.6,0.8} missing rates. Hence, missing values are randomly spread across cases and variables in the dataset. Then, missing values were (once) imputed with the imputation methods mentioned in Section 3.

Although multiple imputation can be very beneficial when analyzing coverage rates for prediction intervals (see, e.g., [41] or [1]) in terms of more accurate uncertainty reflection of the missing mechanism itself, our considered methods, however, are partly limited to be applied within the multiple imputation framework. In [42], for example, the missForest procedure was shown to be not *multiple imputation proper* making its direct usage in the multiple imputation scheme limited. Once missing values are imputed, the whole process is then iterated using $MC_{imp}=500$ Monte-Carlo iterates.

Based on each imputed dataset, all of the above mentioned prediction models are trained and their prediction accuracy is measured using a five-fold cross-validated MSE. Regarding hyper-parameter tuning of the various prediction models, we conducted a grid-search using a ten-fold cross-validation procedure with ten replications on the completely observed data, prior to the generation of missing values. This was conducted using the R-function trainControl of the caret-package [43].

In the second part of our simulation study, synthetic data was generated with missing covariates to detect the effect of imputation accuracy on prediction interval coverage rates. Here, we have focused on point-wise prediction intervals. For sample sizes n∈{100,500,1000}, regression learning problems of the form ${\left\{{\left[X_{i}^{⊤},Y_{i}\right]}^{⊤}\right\}}_{i=1}^{n}$ were generated using a p=10 dimensional covariate space and model (1), where $X_{i}\overset{\mathrm{iid}}{∼}N_{p=10}\left(0,\Sigma \right)$ and $\epsilon_{i}\overset{\mathrm{iid}}{∼}N\left(0,\sigma^{2}\right)$ were simulated independent of each other. Missing values were inserted under the MCAR scheme using various missing rates $r_{PI}\in \left\{0.1,0.2,0.3\right\}$. Regarding the functional relationship between features and response, different regression functions with coefficient $\beta_{0}={\left[2,4,2,-3,1,7,-4,0,0,0\right]}^{⊤}$ were used, such as

1. a linear model: $m\left(x_{i}\right)=x_{i}^{⊤}\beta_{0}$,
2. a polynomial model: $m\left(x_{i}\right)=\sum_{j=1}^{p}\beta_{0,j}x_{i,j}^{j}$,
3. a trigonometric model: $m\left(x_{i}\right)=2\cdot \sin\left(x_{i}^{⊤}\beta_{0}+2\right)$ and
4. a non-continuous model:
  $$\begin{array}{cc} m(x_{i}) & =\left\{\begin{array}{cc} \beta_{0,1}x_{i,1}+\beta_{0,2}x_{i,2}+\beta_{0,3}x_{i,3}, & \;\mathrm{if}\;x_{i,3}>0.5,\\ \beta_{0,4}x_{i,4}+\beta_{0,5}x_{i,5}+3 & \;\mathrm{if}\;x_{i,3}\leq 0.5. \end{array}\right. \end{array}$$

In order to capture potential dependencies among the features, various choices for the covariance matrix Σ were considered: a positive auto-regressive, negative auto-regressive, compound symmetric, Toeplitz and the scaled identity structure. In addition, we aimed to take care of the systematic variation originating from $m(X_{1})$, and the noise $\epsilon_{1}$, by choosing $\sigma^{2}$ in such a way that the signal-to-noise ratio $SN:=Var\left(m\left(X_{1}\right)\right)/\sigma^{2}=1$. Finally, using $MC_{PI}=1000$ Monte-Carlo iterations, prediction interval performance of the intervals proposed in Section 2 are evaluated by approximating coverage rates and (average) interval lengths over the Monte-Carlo iterates.

## 5. Simulation Results

In the sequel, the simulation results for both parts, the empirical datasets obtained through the UCI Machine Learning repository and the simulation study are presented. Note that additional results can be found in Appendix A and in the supplement of [44].

### 5.1. Results on Imputation Accuracy and Model Prediction Accuracy

In this section, we present the results for the empirical data analysis based on the Airfoil dataset using the imputation and prediction accuracy measures described in Section 2 for evaluation. We thereby focus on the Random Forest and the XGBoost prediction model. The results of the linear and the SGB model as well as the results for all other datasets are given in the supplement in [44] (see Figures 1–19 therein) and summarized at the end of this section.

**Figure 1:**
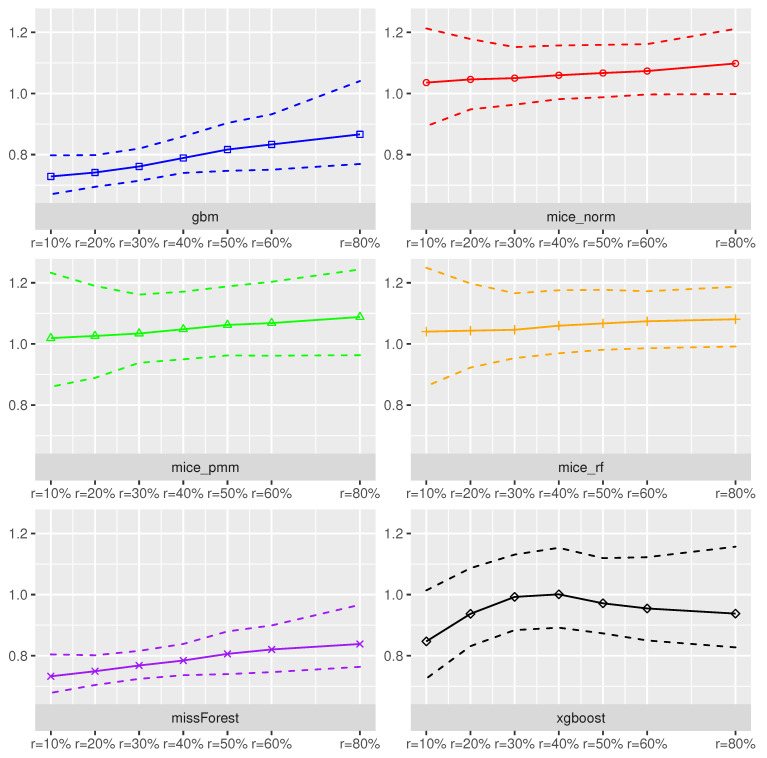
Imputation accuracy measured by NRMSE using the **Random-Forest** method for predicting scaled sound pressure in the **Airfoil dataset** under various missing rates. The dotted lines refer to 95% empirical Monte-Carlo confidence, while the solid lines are Monte-Carlo means of the NRMSE.

**Figure 2:**
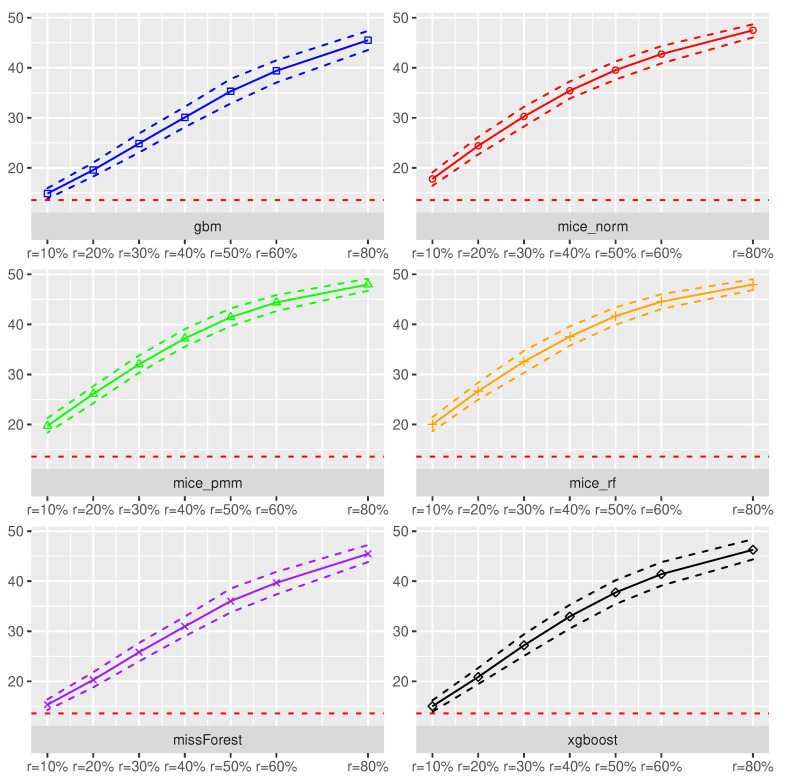
Prediction accuracy measured in MSE using the **Random-Forest** method for predicting scaled sound pressure in the **Airfoil dataset** under various missing rates. The MSE is estimated based on a five-fold cross-validation procedure on the imputed dataset. The dotted lines around the solid curves refer to 95% empirical Monte-Carlo intervals. The solid lines are Monte-Carlo means of the cross-validated MSE. The horizontal dotted line in red refers to the cross-validated MSE of the Random Forest fitted to the Airfoil dataset without any missing values.

**Random Forest as Prediction Model.**Figure 1 and Figure 2 summarize for each imputation method the imputation error (NRMSE) and the model prediction error (MSE) over $MC_{imp}=500$ Monte-Carlo iterates using the Random Forest method for prediction on the imputed dataset. On average, the smallest imputation error measured with the NRMSE could be attained when using missForest and the gbm imputation method. In addition, these methods yielded low variations in NRMSE across the Monte-Carlo iterates. In contrast, the mice_norm, mice_pmm and mice_rf behaved similarly resulting into largest NRMSE values across the different imputation schemes with an increased variation in NRMSE values.

The xgboost method performed slightly worse than missForest and gbm, when focusing on imputation accuracy. In addition, all methods seemed to be more or less robust towards an increased missing rate. Interesting is the fact that volatility decreases, as missing rates increase for the MICE procedures. The prediction accuracy measured in terms of cross-validated MSE using the Random-Forest model was the lowest under the missForest, xgboost and gbm, which corresponds with the NRMSE results.

As expected, the estimated MSE suffered from missing covariates and the effect became worse with an increased missing rate. For example, an increase in the missing rate from 10% to 50% yielded an increase of the NRMSE by 8.4%, while the MSE realized an increase of 127.6%. Hence, model prediction accuracy heavily suffered from an increased amount of missing values, independent of the used imputation scheme. In addition, if the NMRSE increases by 0.1 units, it is expected that the MSE will increase by 122.1%. Although congeniality was defined for valid statistical inference procedures, the effect of using the same method for imputation and prediction seemed to also have a positive effect on model prediction accuracy.

**Figure 3:**
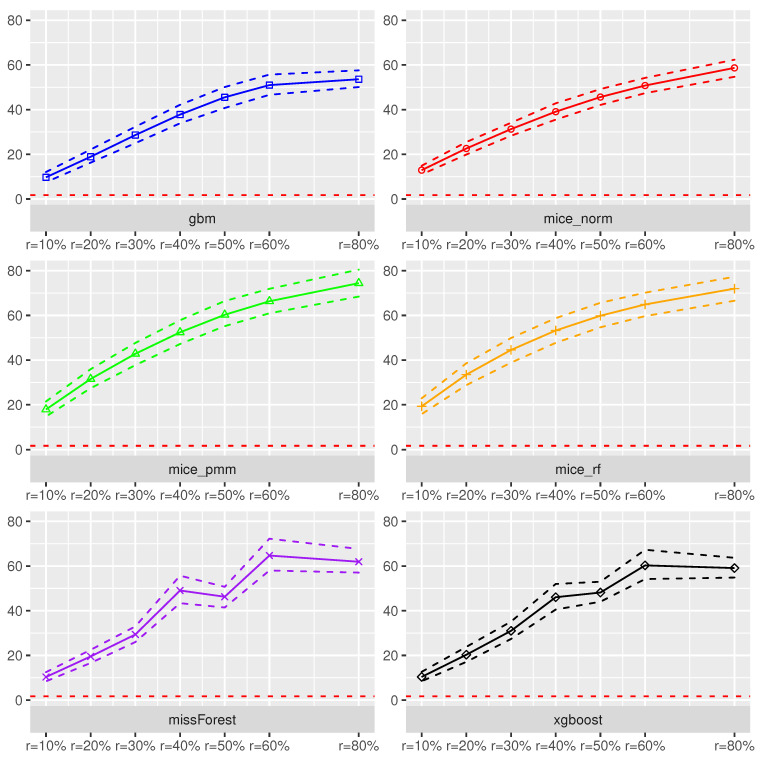
Prediction accuracy measured in MSE using the **XGBoost** method for predicting scaled sound pressure in the **Airfoil dataset** under various missing rates. The MSE is estimated based on a five-fold cross-validation procedure on the imputed dataset. The dotted lines around the solid curves refer to 95% empirical Monte-Carlo intervals. The solid lines are Monte-Carlo means of the cross-validated MSE. The horizontal dotted line in red refers to the cross-validated MSE of the XGBoost method fitted to the Airfoil dataset without any missing values.

**XGBoost as Prediction Model.** In switching the prediction method to XGBoost, we realized an increase in model prediction accuracy for missing rates up to 20% as can be seen in Figure 3. In addition, for those missing rates, the xgboost imputation was competitive to the missForest method but lost in accuracy for larger missing rates compared to the missForest. Different from the Random Forest, the XGBoost prediction method was more sensitive towards an increased missing rate.

For example, an increase of the missing rate from 10% to 50% yields to an increase of the NRMSE by 8.1%, while the MSE suffered by an increase of 300% on average. In addition, an increase of the imputation error by 0.1 points, can yield an average increase of prediction error by 189.9% Although under the completely observed framework the XGBoost method performed best in terms of estimated MSE, the results indicate that missing covariates can disturb the ranking. In fact, for missing rates r≥30%, the Random Forest exhibited a better prediction accuracy.

**Other Prediction models.** Using the linear model as the prediction model resulted in worse prediction accuracy with MSE values ranging from 25 (r=10%) to 45 (r=50%). For all missing scenarios, using the missForest or the gbm method for imputation before prediction with the linear model resulted in the lowest MSE. The results for the SGB method were even worse with MSE values between 80 and 99.

As a surprising result, the prediction accuracy measured in terms of cross-validated MSE decreased with an increasing missing rate. A potential source of this effect could be the general weakness of the SGB in the Airfoil dataset without any missing values. After inserting and imputing missing values, which can yield to distributional changes of the data, it seems that the SGB method benefits from these effects. However, model prediction accuracy is still not satisfactory, see Figure 2 in the supplement in [44].

**Figure 4:**
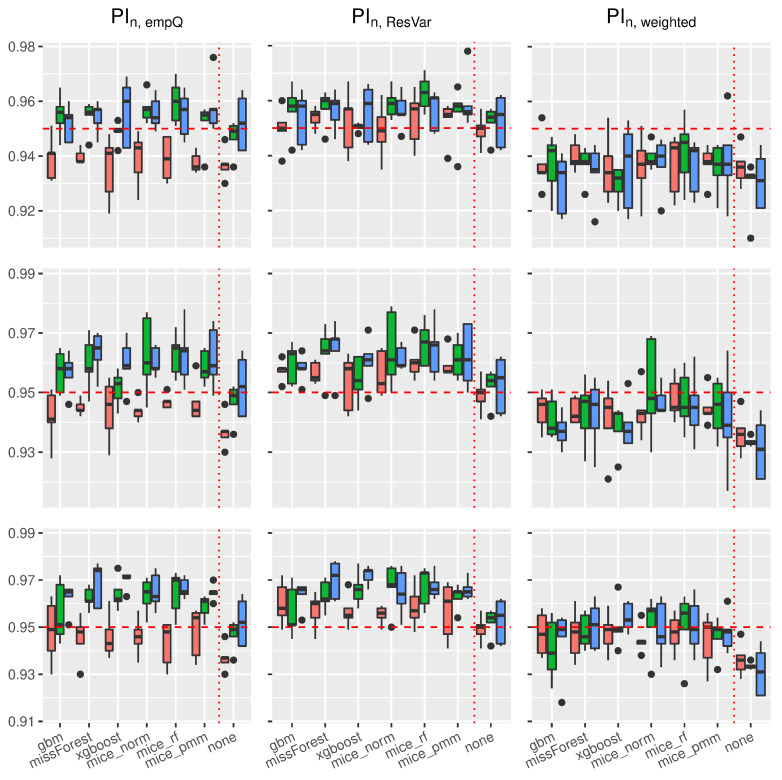
Boxplots of prediction **coverage rates** under the **linear model**. The variation is over the different covariance structures of the features. Each row corresponds to one of the missing rates r∈{0.1,0.2,0.3}, while each column corresponds to the following prediction intervals: $PI_{n,empQ}$, $PI_{n,ResVar}$ and $PI_{n,weighted}$. The triple (red, green and blue) correspond to the sample sizes n∈(100,500,1000).

**Other Datasets.** For the other datasets, similar effects were obtained. The Random Forest and the XGBoost showed the best prediction accuracy, see Figures 4–19 in the supplement in [44]. Again, larger missing rates affected model prediction accuracy for the XGBoost method, but the Random Forest was more robust to them overcoming XGBoost prediction performance measured in cross-validated MSE for larger missing rates. Overall, NRMSE and cross-validated MSE seem to be positively associated to each other. Hence, more accurate imputation models seemed to yield better model prediction measured by MSE.

### 5.2. Results on Prediction Coverage and Length

Using the prediction intervals in Section 2, we present coverage rates and interval lengths of point-wise prediction intervals in simulated data. Both quantities were computed using 1000 Monte-Carlo iterations with sample sizes n∈{100,500,1000}. The boxplots presented here (see Figure 4, Figure 5, Figure 6 and Figure 7) and in the supplement in [44] (see Figures 19–30 therein) spread over the different covariance structures used during the simulation. Every row corresponds to one of the simulated missing rates r∈{0.1,0.2,0.3}, while the columns reflect the different Random-Forest-based prediction intervals.

The left column summarizes the results for the Random-Forest-based prediction interval using empirical quantiles ($PI_{n,empQ}$), the center column reflects the Random Forest prediction interval using the simple residual variance estimator on Out-of-Bag errors ($PI_{n,ResVar}$), while the right column summarizes the Random-Forest-based prediction interval using the weighted residual variance estimator ($PI_{n,weighted}$). We shifted the results of $PI_{QRF,n}$, $PI_{n,MCorrect}$ and the prediction interval based on the linear model to the supplement in [44] (see Figures 19–22, 24, 26, 28 and 30 therein). Under the complete case scenarios, the latter methods did not show comparably well coverage rates as $PI_{n,emQ}$, $PI_{n,ResVar}$ and $PI_{n,weighted}$. For imputed missing covariates, the methods performed with less accuracy in terms of correct coverage rates, when comparing them with $PI_{n,emQ}$, $PI_{n,ResVar}$ and $PI_{n,weighted}$.

Although the interval lengths of $PI_{QRF,n}$, $PI_{n,MCorrect}$ and the linear model were, on average smaller, the coverage rate was not sufficient to make them competitive with $PI_{n,emQ}$, $PI_{n,ResVar}$ and $PI_{n,weighted}$. For prediction intervals that underestimated the 0.95 threshold in the complete case scenario, we observed more accurate coverage rates for larger missing rates. It seems that larger missing rates increase coverage rates for the $PI_{QRF,n}$ and $PI_{n,MCorrect}$ methods, independent of the used imputation scheme.

In Figure 4, the boxplots of the linear regression model are presented. In general, the use of Random-Forest-based prediction intervals with empirical quantiles ($PI_{n,empQ}$) or simple variance estimation ($PI_{n,ResVar}$) show competitive behavior in the complete case scenario. When considering the various imputation schemes, under the different missing rates, it can be seen that coverage rate slightly suffered compared to the complete case. To be more precise, larger missing rates lead to slightly larger coverage rates for $PI_{n,emQ}$, $PI_{n,ResVar}$ and $PI_{n,weighted}$.

For the Random-Forest-based prediction interval with weighted residual variance, this effect seems to be positive, i.e., larger missing rates will lead to better coverage rates for $PI_{n,weighted}$. Comparing the results with the previous findings, we see that the xgboost yields, on average, the best coverage results across the different imputation schemes. While the MICE procedures did not reveal competitive performance in model prediction accuracy, the mice_norm method under the linear model performed similar to the missForest procedure when comparing coverage rates.

**Figure 5:**
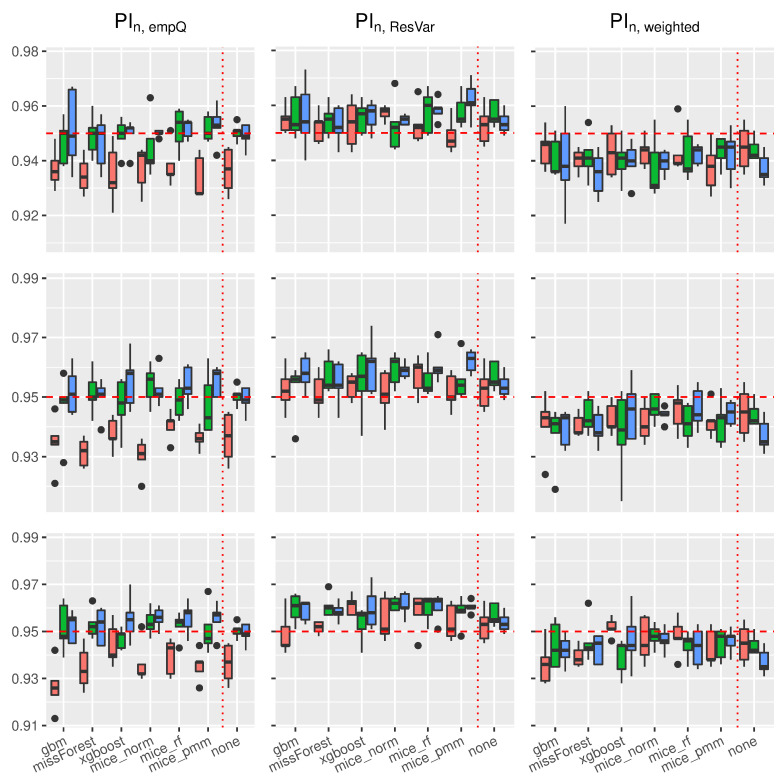
Boxplots of prediction **coverage rates** under the **trigonometric model**. The variation is over the different covariance structures of the features. Each row corresponds to one of the missing rates r∈{0.1,0.2,0.3}, while each column corresponds to the following prediction intervals: $PI_{n,empQ}$, $PI_{n,ResVar}$ and $PI_{n,weighted}$. The triple (red, green and blue) correspond to the sample sizes n∈(100,500,1000).

Figure 5 summarizes coverage rates of point-wise prediction intervals under the trigonometric model. Similar to the linear case, all three methods $PI_{n,empQ}$, $PI_{n,ResVar}$ and $PI_{n,weighted}$ yielded accurate coverage rates showing better approximation to the 0.95 threshold when the sample size increase under the complete observation case. On average, the xgboost imputation method remains competitive compared to the other imputation methods.

Slightly different from the linear case, the mice_norm approach gains in correct coverage rate approximation compared to the missForest, together with the mice_pmm approach. Nevertheless, the approximations between mice_norm, mice_pmm and missForest are close to each other. As mentioned earlier, $PI_{n,weighted}$ turns more accurate in terms of correct coverage rates, when the missing rate increases. Similar results compared to the linear and trigonometric case could be obtained for the polynomial model and the non-continuous model. Boxplots of the coverage rates can be found in Figures 24 and 28 of the supplement in [44].

**Figure 6:**
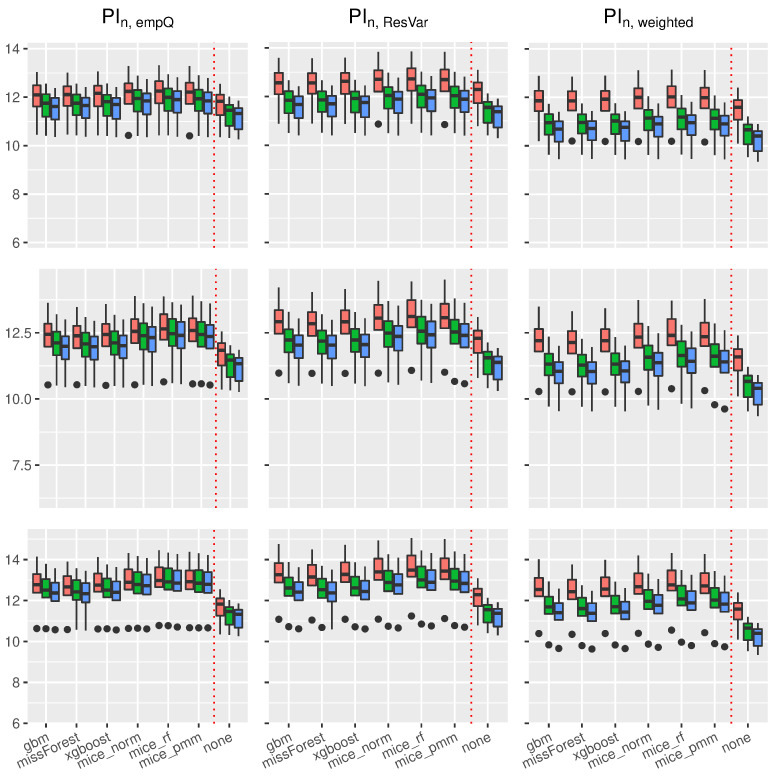
Boxplots of prediction **interval lengths** under the **linear model**. The variation is over the different covariance structures of the features. Each row corresponds to one of the missing rates r∈{0.1,0.2,0.3}, while each column corresponds to the following prediction intervals: $PI_{n,empQ}$, $PI_{n,ResVar}$ and $PI_{n,weighted}$. The triple (red, green and blue) correspond to the sample sizes n∈(100,500,1000).

Regarding the length of the intervals for the linear model (see Figure 6), the prediction interval $PI_{n,empQ}$ and the parametric interval $PI_{n,ResVar}$ yielded similar interval lengths. Under imputed missing covariates, however, the $PI_{n,empQ}$ interval led to slightly smaller intervals than $PI_{n,ResVar}$. Nevertheless, the prediction interval based on the weighted residual variance estimator $PI_{n,wighted}$ had the smallest intervals on average. This comes with the cost of less accurate coverage rates as can be seen in Figure 4.

In addition, independent of the used prediction interval, an increased missing rate yielded larger intervals making the learning methods, such as Random Forest, more insecure about future predictions. Regarding the used imputation method, almost all imputation methods resulted in similar interval lengths. On average, the missForest method had slightly smaller intervals comparable to the xgboost imputation.

**Figure 7:**
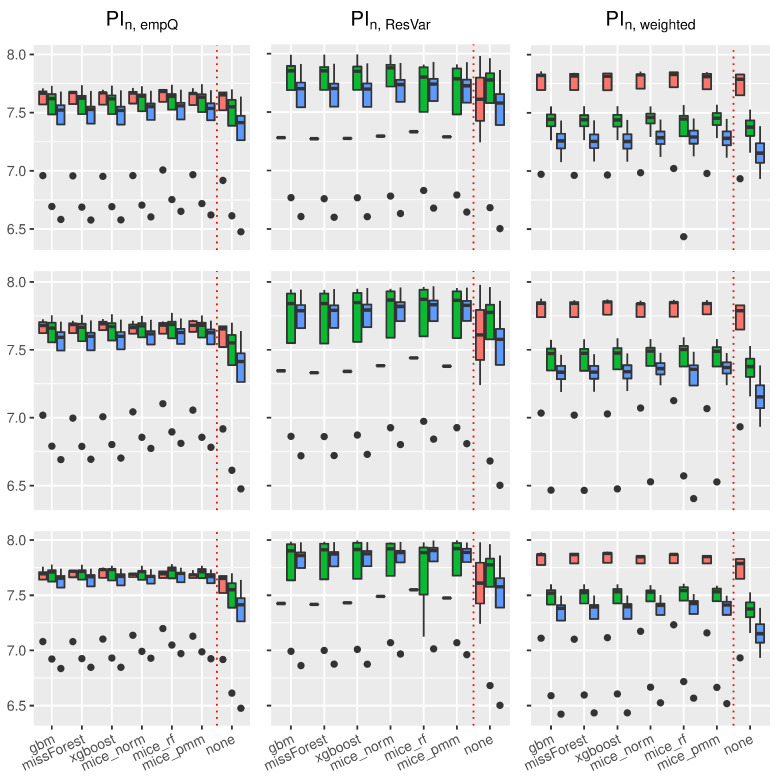
Boxplots of prediction **interval lengths** under the **trigonometric model**. The variation is over the different covariance structures of the features. Each row corresponds to one of the missing rates r∈{0.1,0.2,0.3}, while each column corresponds to the following prediction intervals: $PI_{n,empQ}$, $PI_{n,ResVar}$ and $PI_{n,weighted}$. The triple (red, green and blue) correspond to the sample sizes n∈(100,500,1000).

Similar results on prediction lengths were obtained with other models. Considering the trigonometric function as in Figure 7, it can be seen that $PI_{n,empQ}$ results in slightly smaller intervals than $PI_{n,ResVar}$. However, the interval lengths for the empirical quantiles under the trigonometric model were more robust towards dependent covariates.

Comparably to the linear case, $PI_{n,weighted}$ results in the smallest interval lengths, but suffers from less accurate coverage. Furthermore, all imputation methods behave similar with respect to prediction interval lengths under the trigonometric case and other models (see Figures 21 and 22 in the supplement in [44]). It can be seen that Random-Forest-based prediction intervals are, more or less, universally applicable to the different imputation schemes used in this scenario yielding similar interval lengths.

In summary, Random-Forest-based prediction intervals with imputed missing covariates yielded slightly wider intervals compared to the regression framework without missing values. For prediction intervals that underestimated the true coverage rate, such as $PI_{QRF,n}$, $PI_{n,MCorrect}$ and $PI_{n,weighted}$, an increased missing rate had positive effects on the coverage rate. Overall, missForest and xgboost were competitive imputation schemes when considering accurate coverage rates and interval lengths using the $PI_{n,empQ}$ and $PI_{n,ResVar}$ intervals. mice_norm resulted in similar, but slightly less accurate, coverage compared to missForest and xgboost.

## 6. Conclusions

Missing covariates in regression learning problems are common issues in data analysis problems. Providing a general approach for enabling the application of various analysis models is often obtained through imputation. The use of ML-based methods in this framework has obtained increased attention over the last decade, since fast and easy to use ML methods, such as the Random Forest, can provide us with quick and accurate fixes for data analysis problems.

In our work, we placed a special focus on variants of ML-based methods for imputation, which mainly rely on decision trees as base learners, and their aggregation is conducted in a Random-Forest-based fashion or a boosting approach. We aimed to shed light into the general issue when and which imputation method should be used for missing covariates in regression learning problems that provide accurate point predictions with correct uncertainty ranges. To provide an answer to this, we conducted empirical analyses and simulations, which were led by the following questions:

Does an imputation scheme with a low imputation error (measured with the NRMSE) automatically provide us with accurate model prediction performance (in terms of cross-validated MSE)? How do ML-based imputation methods perform in estimating uncertainty ranges for future prediction points in form of point-wise prediction intervals? Are the results in harmony; that is, does an accurate imputation method with a low NRMSE provide us with good model prediction accuracy measured in MSE while delivering accurate and narrow prediction interval lengths?

By analyzing empirical data from the UCI Machine Learning repository, we found that imputation methods with low imputation error measured with the NRMSE yielded better model prediction measured by cross-validated MSE. In our analysis, we could see that an increased missing rate had a negative effect on both the MSE and the NRMSE, while on the latter, the effect was less expressive. Particularly, for larger missing rates, the use of the same ML method for both imputation and prediction was beneficial. This is in line with the *congeniality* assumption; a theoretical term that (partly) guarantees correct inference after (multiple) imputation.

In particular, the missForest and our modified xgboost method for imputation yielded preferable results in terms of a low imputation error and good model prediction. It is expected that ML methods with accurate model prediction capabilities measured in MSE can be transformed to be used as an imputation method yielding low imputation errors as well. Regarding statistical inference procedures in prediction settings, such as the construction of valid prediction intervals, Random-Forest-based imputation schemes, such as the missForest and the xgboost method, yielded competitive coverage rates and interval lengths.

In addition, the MICE procedure with a Bayesian linear regression and normal assumption was under the aspect of correct coverage rates and interval lengths competitive as well. However, the method did not reveal low imputation error and overall good model prediction.

Hence, based on our findings, the missForest and the xgboost method in combination with Random-Forest-based prediction intervals using empirical quantiles resp. Out-of-Bag estimated residual variances are competitive in three aspects: providing low imputation errors measured with the NRMSE, yielding comparably low model prediction errors measured by cross-validated MSE and providing comparably accurate prediction interval coverage rates and narrow widths using Random-Forest-based intervals. Regarding the latter, our results also indicate that these intervals are competitively applicable to a wide range of imputation schemes.

In summary, data analysts that fully rely on prediction accuracy after imputing missing data should focus on imputation schemes with comparably low NRMSE as a prior indicator, especially when using tree-based ML methods. In addition, the same or more general imputation methods should be used. However, when moving to predictive statistical inference in terms of accurate prediction coverage rates, the NRMSE is not a direct measure indicating good coverage results.

Future research will focus on a theoretical exploration of the interaction between the NRMSE and MSE and the effect of the considered imputation methods on uncertainty estimators in multiple imputation scenarios. The aim is to discover the type of impact several factors have on the interactions between both measures, such as the missing rate, the missing structure and the used prediction method on more general imputation schemes accounting for multiple imputation as well. Insights into their theoretical interaction will provide additional information to the general issue that *imputation is not only prediction.*

## Data Availability

This work contains data extracted from the UCI Machine Learning Repository https://archive.ics.uci.edu/ml/index.php (accessed on 19 October 2021). In addition, simulation-based data is used in this work as well. The simulation design and procedure is described in Section 4 in detail.

## Appendix A. Supplementary Results

This work contains supplementary material amending additional simulation results on the empirical and simulation based analysis. Due to its extensive length, supplemental results are shifted to the arXiv version of this work and can be found in supplementary materials of [44].

### Appendix A.1. Descriptive Statistics

Regarding the empirical data used from the UCI Machine Learning Repository, we provide summary statistics for all five datasets. The tables can be extracted in the following:

**Table A1:** Summary statistics of the Real Estate Dataset.

Real Estate Dataset				
Variable	Scales of Measurement	Range	Mean/Median	Variance/IQR
Transaction Date	ordinal	between 2012 & 2013	−−/−−	−−/−−
House Price per m^2^	continuous	[7.6;117.5]	37.98/38.45	185.14/18.9
House Age	continuous	[0;43.8]	17.71/16.1	129.79/19.13
Distance to the nearest MRT station	continuous	[23.38;6488.02]	1083.87/492.23	1,592,921/1164.95
Coordinate (latitude)	continuous	[24.93;25.01]	24.97/24.97	0.00015/0.0144
Coordinate (longitude)	continuous	[121.47;121.56]	121.53/121.54	0.00026/0.015

**Table A2:** Summary statistics of the Airfoil Dataset.

Airfoil Dataset				
Variable	Scales of Measurement	Range	Mean/Median	Variance/IQR
Scaled Sound Pressure	continuous	[103.38;140.99]	124.84/125.72	47.59/9.80
Frequency	discrete—ordinal	$\begin{array}{cc} & \{200,250,315,400,500,\\ & 630,800,1000,1250,\\ & 1600,2000,2500,2500,\\ & 3150,4000,5000,6300,\\ & 8000,10,000,12,500,\\ & 16,000,20,000\} \end{array}$	−−/1600	−−/−−
Angle of Attack	discrete—ordinal	$\begin{array}{cc} & \{0,1.5,2,2.7,3,3.3,4,4.2,\\ & 4.8,5.3,5.4,6.7,7.2,7.2,\\ & 7.3,8.4,8.9,9.5,9.9,11.2,\\ & 12.3,12.6,12.7,15.4,15.6,\\ & 17.4,19.7,22.2\} \end{array}$	−−/5.4	−−/−−
Chord length	discrete—ordinal	$\begin{array}{cc} & \{0.0254,0.0508,0.1016,\\ & 0.1524,0.2286,0.3048\} \end{array}$	−−/0.1016	−−/−−
Free-stream velocity	discrete—ordinal	$\begin{array}{cc} & \left\{31.7,39.6,55.5,71.3\right\} \end{array}$	−−/39.6	−−/−−
Suction side displacement thickness	continuous	$\begin{array}{cc} & \left[0.000400682;0.0584113\right] \end{array}$	0.01113988/ 0.00495741	0.00017/0.01304

**Table A3:** Summary statistics of the Power Plant Dataset.

Power Plant Dataset				
Variable	Scales of Measurement	Range	Mean/Median	Variance/IQR
Electric Energy Output	continuous	[420.26;495.76]	454.37/451.55	291.28/28.68
Temperature	continuous	[1.91;37.11]	19.65/20.35	55.54/12.21
Exhaust Vaccuum	continuous	[25.36;81.56]	54.31/52.08	161.49/24.8
Ambient Pressure	continuous	[992.89;1033.3]	1013.26/1012.94	35.27/8.16
Relative Humidity	continuous	[25.56;100.16]	73.31/74.98	213.17/21.50

**Table A4:** Summary statistics of the Concrete Dataset.

Concrete Dataset				
Variable	Scales of Measurement	Range	Mean/Median	Variance/IQR
Compressive Strength	continuous	[2.33;82.6]	35.82/34.45	279.08/22.43
Cement Component	continuous	[102;540]	281.17/272.9	10,921.58/157.625
Blast Furnance Slag Component	continuous	[0;359.4]	73.90/22	7444.125/142.95
Fly Ash Component	continuous	[0;200.1]	54.19/0	4095.62/118.3
Water Component	continuous	[121.8;247]	181.57/185	456/27.1
Super- plasticizer	continuous	[0;32.2]	6.205/6.4	35.67/10.2
Coarse Aggregate Component	continuous	[801;1145]	972.92/968	6045.68/97.4
Fine Aggregate Component	continuous	[594;992.6]	773.58/779.5	6428.19/93.05
Age in Days	continuous	[1;365]	46.66/28	3990.44/49

**Table A5:** Summary statistics of the QSAR Dataset, after removing nominal variables. These are *H-050*, *nN* and *C-040*.

QSAR Dataset				
Variable (Molecular Description)	Scales of Measurement	Range	Mean/Median	Variance/IQR
LC50	continuous	[0.12;10.05]	4.66/4.52	2.73/2.01
TPSA(Tot)	continuous	[0;347.32]	48.47/40.46	2186.87/54.23
SAacc	continuous	[0;571.95]	58.87/42.68	4646.68/66.49
MLOGP	continuous	[−6.45;9.15]	2.31/2.27	3.03/2.16
RDCHI	continuous	[1;6.46]	2.49/2.34	0.66/0.94
GATS1p	continuous	[0.28;2.5]	1.05/1.02	0.163/0.53

### Appendix A.2. More Detailed Results

We furthermore provide tables for Figure 1, Figure 2, Figure 3, Figure 4, Figure 5, Figure 6 and Figure 7, covering both imputation error and prediction coverage rates.

#### Appendix A.2.1. Imputation and Prediction Error

**Table A6:** Monte-Carlo mean of the NMRSE for the **Airfoil Dataset** summarizing the same information as in Figure 1.

Mean Monte-Carlo NRMSE of the Airfoil Dataset							
Imputation Method	r=10%	r=20%	r=30%	r=40%	r=50%	r=60%	r=80%
missForest	0.729	0.741	0.762	0.782	0.805	0.819	0.841
mice_pmm	1.028	1.032	1.039	1.043	1.054	1.065	1.093
mice_norm	1.046	1.044	1.049	1.059	1.063	1.075	1.098
mice_rf	1.040	1.042	1.054	1.058	1.068	1.072	1.080
gbm	0.724	0.740	0.759	0.789	0.813	0.830	0.869
xgboost	0.843	0.939	0.992	1.001	0.978	0.951	0.940

**Table A7:** Monte-Carlo mean of the MSE for the **Airfoil Dataset** summarizing the same information as in Figure 2 Using the Random Forest prediction method.

Mean Monte-Carlo MSE of the Airfoil Dataset Using Random Forest							
Prediction Method	r=10%	r=20%	r=30%	r=40%	r=50%	r=60%	r=80%
missForest	15.339	20.288	25.810	30.974	36.024	39.679	45.458
mice_pmm	19.722	26.118	32.024	37.191	41.433	44.384	47.487
mice_norm	17.799	24.411	30.271	35.412	39.534	42.735	58.684
mice_rf	20.029	26.667	32.581	37.550	41.662	44.541	47.972
gbm	14.881	19.576	24.870	30.091	35.295	39.387	45.498
xgboost	15.021	20.866	27.193	32.960	37.745	41.412	46.276
Fully observed	13.582						

**Table A8:** Monte-Carlo mean of the MSE for the **Airfoil Dataset** summarizing the same information as in Figure 2 Using the XGBoost prediction method.

Mean Monte-Carlo MSE of the Airfoil Dataset Using XGBoost							
Prediction Method	r=10%	r=20%	r=30%	r=40%	r=50%	r=60%	r=80%
missForest	10.342	19.538	29.366	49.054	46.262	64.705	61.934
mice_pmm	17.886	31.541	42.823	52.425	60.276	66.325	74.428
mice_norm	12.910	22.596	31.275	39.078	45.623	50.780	58.684
mice_rf	19.303	33.487	44.539	53.246	59.839	64.895	71.973
gbm	9.705	18.905	28.625	37.793	45.518	50.977	53.598
xgboost	10.383	20.300	31.008	46.056	48.145	60.292	59.109
Fully observed	1.691						

#### Appendix A.2.2. Prediction Coverage Rates

**Table A9:** Triple of simulated prediction coverage rates averaged over the five different covariance structures for the linear model and r=10% missing rates. The triple covers the sample sizes $\left(n_{1};n_{2};n_{3}\right)=\left(100,500,1000\right)$ using a significance level of α=0.05.

Coverage Rate for the Linear Model with *r* = 10% Missings			
Imputation Method	$PI_{n,empQ}$	$PI_{n,ResVar}$	$PI_{n,weighted}$
missForest	(0.9398;0.9544;0.9542)	(0.9538;0.9574;0.9564)	(0.9402;0.9370;0.9340)
mice_pmm	(0.9376;0.9514;0.9584)	(0.9526;0.9548;0.9598)	(0.9372;0.9356;0.9388)
mice_norm	(0.9390;0.9574;0.9558)	(0.9490;0.9590;0.9564)	(0.9360;0.9396;0.9372)
mice_rf	(0.9390;0.9598;0.9552)	(0.9534;0.9628;0.9564)	(0.9368;0.9416;0.9360)
gbm	(0.9392;0.9550;0.9518)	(0.9498;0.9568;0.9536)	(0.9370;0.9368;0.9298)
xgboost	(0.9356;0.9486;0.9560)	(0.9524;0.9504;0.9556)	(0.9356;0.9298;0.9358)
Fully observed	(0.9370;0.9468;0.9522)	(0.9492;0.9522;0.9526)	(0.9362;0.9288;0.9312)

**Table A10:** Triple of simulated prediction coverage rates averaged over the five different covariance structures for the linear model and r=20% missing rates. The triple covers the sample sizes $\left(n_{1};n_{2};n_{3}\right)=\left(100,500,1000\right)$ using a significance level of α=0.05.

Coverage Rate for the Linear Model with *r* = 20% Missings			
Imputation Method	$PI_{n,empQ}$	$PI_{n,ResVar}$	$PI_{n,weighted}$
missForest	(0.9450;0.9598;0.9634)	(0.9566;0.9634;0.9646)	(0.9438;0.9434;0.9430)
mice_pmm	(0.9468;0.9586;0.9618)	(0.9596;0.9614;0.9622)	(0.9450;0.9448;0.9410)
mice_norm	(0.9440;0.9626;0.9604)	(0.9562;0.9646;0.9616)	(0.9436;0.9516;0.9472)
mice_rf	(0.9470;0.9628;0.9628)	(0.9606;0.9664;0.9644)	(0.9480;0.9474;0.9452)
gbm	(0.9418;0.9570;0.9566)	(0.9574;0.9598;0.9582)	(0.9440;0.9414;0.9372)
xgboost	(0.9440;0.9514;0.9598)	(0.9534;0.9546;0.9604)	(0.9412;0.9384;0.9392)

**Table A11:** Triple of simulated prediction coverage rates averaged over the five different covariance structures for the linear model and r=30% missing rates. The triple covers the sample sizes $\left(n_{1};n_{2};n_{3}\right)=\left(100,500,1000\right)$ using a significance level of α=0.05.

Coverage Rate for the Linear Model with *r* = 30% Missings			
Imputation Method	$PI_{n,empQ}$	$PI_{n,ResVar}$	$PI_{n,weighted}$
missForest	(0.9454;0.9624;0.9684)	(0.9570;0.9636;0.9700)	(0.9468;0.9482;0.9506)
mice_pmm	(0.9478;0.9586;0.9648)	(0.9570;0.9628;0.9660)	(0.9444;0.9460;0.9478)
mice_norm	(0.9460;0.9634;0.9654)	(0.9552;0.9672;0.9648)	(0.9448;0.9514;0.9490)
mice_rf	(0.9430;0.9646;0.9666)	(0.9588;0.9674;0.9674)	(0.9474;0.9508;0.9506)
gbm	(0.9482;0.9562;0.9622)	(0.9602;0.9568;0.9634)	(0.9472;0.9406;0.9440)
xgboost	(0.9460;0.9642;0.9698)	(0.9568;0.9662;0.9720)	(0.9476;0.9508;0.9542)

**Table A12:** Triple of simulated prediction coverage rates averaged over the five different covariance structures for the trigonometric model and r=10% missing rates. The triple covers the sample sizes $\left(n_{1};n_{2};n_{3}\right)=\left(100,500,1000\right)$ using a significance level of α=0.05.

Coverage Rate for the Trigonometric Model with *r* = 10% Missings			
Imputation Method	$PI_{n,empQ}$	$PI_{n,ResVar}$	$PI_{n,weighted}$
missForest	(0.9348;0.9492;0.9466)	(0.9522;0.9546;0.9528)	(0.9402;0.9416;0.9352)
mice_pmm	(0.9338;0.9518;0.9530)	(0.9490;0.9578;0.9620)	(0.9376;0.9434;0.9424)
mice_norm	(0.9372;0.9450;0.9500)	(0.9572;0.9526;0.9548)	(0.9432;0.9374;0.9394)
mice_rf	(0.9382;0.9516;0.9518)	(0.9530;0.9578;0.9584)	(0.9434;0.9420;0.9424)
gbm	(0.9372;0.9470;0.9516)	(0.9550;0.9564;0.9562)	(0.9444;0.9410;0.9396)
xgboost	(0.9350;0.9492;0.9494)	(0.9534;0.9556;0.9562)	(0.9430;0.9402;0.9396)
none	(0.9364;0.9504;0.9490)	(0.9528;0.9570;0.9538)	(0.9448;0.9442;0.9372)

**Table A13:** Triple of simulated prediction coverage rates averaged over the five different covariance structures for the trigonometric model and r=20% missing rates. The triple covers the sample sizes $\left(n_{1};n_{2};n_{3}\right)=\left(100,500,1000\right)$ using a significance level of α=0.05.

Coverage Rate for the Trigonometric Model with *r* = 20% Missings			
Imputation Method	$PI_{n,empQ}$	$PI_{n,ResVar}$	$PI_{n,weighted}$
missForest	(0.9314;0.9516;0.9498)	(0.9512;0.9574;0.9546)	(0.9404;0.9440;0.9396)
mice_pmm	(0.9362;0.9476;0.9554)	(0.9518;0.9558;0.9620)	(0.9420;0.9416;0.9446)
mice_norm	(0.9300;0.9542;0.9528)	(0.9510;0.9594;0.9590)	(0.9414;0.9464;0.9440)
mice_rf	(0.9404;0.9488;0.9542)	0.9572;0.9558;0.9606)	(0.9456;0.9412;0.9462)
gbm	(0.9346;0.9466;0.9520)	0.9526;0.9526;0.9582)	(0.9408;0.9372;0.9396)
xgboost	(0.9376;0.9472;0.9556)	(0.9536;0.9550;0.9604)	(0.9428;0.9378;0.9456)

**Table A14:** Triple of simulated prediction coverage rates averaged over the five different covariance structures for the trigonometric model and r=30% missing rates. The triple covers the sample sizes $\left(n_{1};n_{2};n_{3}\right)=\left(100,500,1000\right)$ using a significance level of α=0.05.

Coverage Rate for the Trigonometric Model with *r* = 30% Missings			
Imputation Method	$PI_{n,empQ}$	$PI_{n,ResVar}$	$PI_{n,weighted}$
missForest	(0.9346;0.9530;0.9522)	(0.9516;0.9608;0.9584)	(0.9394;0.9460;0.9426)
mice_pmm	(0.9354;0.9506;0.9552)	(0.9538;0.9582;0.9604)	(0.9428;0.9448;0.9460)
mice_norm	(0.9364;0.9540;0.9558)	(0.9554;0.9618;0.9614)	(0.9464;0.9480;0.9464)
mice_rf	(0.9394;0.9524;0.9558)	(0.9582;0.9598;0.9604)	(0.9480;0.9440;0.9434)
gbm	(0.9266;0.9518;0.9520)	(0.9490;0.9598;0.9588)	(0.9366;0.9442;0.9420)
xgboost	(0.9444;0.9470;0.9554)	(0.9612;0.9532;0.9600)	(0.9518;0.9392;0.9468)

#### Appendix A.2.3. Prediction Interval Length

**Table A15:** Triple of simulated prediction interval lengths averaged over the five different covariance structures for the linear model and r=10% missing rates. The triple covers the sample sizes $\left(n_{1};n_{2};n_{3}\right)=\left(100,500,1000\right)$ using a significance level of α=0.05.

Prediction Interval Length for the Linear Model with *r* = 10% Missings			
Imputation Method	$PI_{n,empQ}$	$PI_{n,ResVar}$	$PI_{n,weighted}$
missForest	(11.934;11.622;11.512)	(12.421;11.726;11.556)	(11.723;10.827;10.571)
mice_pmm	(12.044;11.794;11.701)	(12.540;11.899;11.744)	(11.841;10.998;10.758)
mice_norm	(12.048;11.787;11.677)	(12.540;11.884;11.718)	(11.841;10.985;10.733)
mice_rf	(12.081;11.831;11.740)	(12.576;11.927;11.775)	(11.875;11.025;10.787)
gbm	(11.938;11.609;11.479)	(12.423;11.713;11.526)	(11.725;10.814;10.542)
xgboost	(11.951;11.622;11.506)	(12.437;11.733;11.553)	(11.739;10.834;10.568)
none	(11.620;11.261;11.133)	(12.097;11.360;11.184)	(11.405;10.467;10.204)

**Table A16:** Triple of simulated prediction interval lengths averaged over the five different covariance structures for the linear model and r=20% missing rates. The triple covers the sample sizes $\left(n_{1};n_{2};n_{3}\right)=\left(100,500,1000\right)$ using a significance level of α=0.05.

Prediction Interval Length for the Linear Model with *r* = 20% Missings			
Imputation Method	$PI_{n,empQ}$	$PI_{n,ResVar}$	$PI_{n,weighted}$
missForest	(12.214;11.953;11.840)	(12.701;12.053;11.886)	(11.997;11.149;10.896)
mice_pmm	(12.457;12.315;12.237)	(12.969;12.411;12.276)	(12.263;11.503;11.281)
mice_norm	(12.420;12.253;12.160)	(12.930;12.344;12.201)	(12.226;11.440;11.210)
mice_rf	(12.519;12.347;12.267)	(13.017;12.439;12.297)	(12.310;11.529;11.301)
gbm	(12.283;12.001;11.857)	(12.785;12.099;11.903)	(12.079;11.194;10.913)
xgboost	(12.269;12.003;11.875)	(12.769;12.102;11.918)	(12.063;11.196;10.927)

**Table A17:** Triple of simulated prediction interval lengths averaged over the five different covariance structures for the linear model and r=30% missing rates. The triple covers the sample sizes $\left(n_{1};n_{2};n_{3}\right)=\left(100,500,1000\right)$ using a significance level of α=0.05.

Prediction Interval Length for the Linear Model with *r* = 30% Missings			
Imputation Method	$PI_{n,empQ}$	$PI_{n,ResVar}$	$PI_{n,weighted}$
missForest	(12.523;12.323;12.219)	(13.035;12.419;12.264)	(12.325;11.507;11.267)
mice_pmm	(12.800;12.745;12.690)	(13.335;12.841;12.726)	(12.622;11.926;11.725)
mice_norm	(12.784;12.669;12.598)	(13.298;12.759;12.628)	(12.587;11.849;11.633)
mice_rf	(12.899;12.826;12.756)	(13.434;12.922;12.793)	(12.720;12.004;11.789)
gbm	(12.646;12.421;12.273)	(13.166;12.515;12.312)	(12.453;11.600;11.313)
xgboost	(12.648;12.428;12.310)	(13.171;12.526;12.348)	(12.456;11.610;11.348)

**Table A18:** Triple of simulated prediction interval lengths averaged over the five different covariance structures for the trigonometric model and r=10% missing rates. The triple covers the sample sizes $\left(n_{1};n_{2};n_{3}\right)=\left(100,500,1000\right)$ using a significance level of α=0.05.

Prediction Interval Length for the Trigonometric Model with *r* = 10% Missings			
Imputation Method	$PI_{n,empQ}$	$PI_{n,ResVar}$	$PI_{n,weighted}$
missForest	(7.514;7.436;7.349)	(7.960;7.636;7.501)	(7.630;7.213;7.039)
mice_pmm	(7.515;7.451;7.373)	(7.964;7.654;7.530)	(7.635;7.231;7.067)
mice_norm	(7.520;7.448;7.372)	(7.969;7.654;7.530)	(7.640;7.231;7.067)
mice_rf	(7.535;7.463;7.386)	(7.983;7.668;7.545)	(7.653;7.243;7.081)
gbm	(7.517;7.438;7.350)	(7.965;7.639;7.502)	(7.635;7.216;7.041)
xgboost	(7.512;7.436;7.347)	(7.960;7.638;7.502)	(7.631;7.215;7.040)
none	(7.488;7.372;7.253)	(7.934;7.566;7.397)	(7.606;7.146;6.941)

**Table A19:** Triple of simulated prediction interval lengths averaged over the five different covariance structures for the trigonometric model and r=20% missing rates. The triple covers the sample sizes $\left(n_{1};n_{2};n_{3}\right)=\left(100,500,1000\right)$ using a significance level of α=0.05.

Prediction Interval Length for the Trigonometric Model with *r* = 20% Missings			
Imputation Method	$PI_{n,empQ}$	$PI_{n,ResVar}$	$PI_{n,weighted}$
missForest	(7.543;7.493;7.427)	(7.995;7.700;7.590)	(7.664;7.274;7.123)
mice_pmm	(7.560;7.516;7.463)	(8.007;7.725;7.634)	(7.676;7.298;7.165)
mice_norm	(7.545;7.514;7.458)	(8.002;7.725;7.629)	(7.671;7.299;7.162)
mice_rf	(7.562;7.529;7.474)	(8.019;7.742;7.643)	(7.687;7.314;7.174)
gbm	(7.550;7.492;7.423)	(8.000;7.700;7.588)	(7.669;7.274;7.122)
xgboost	(7.555;7.500;7.432)	(8.006;7.708;7.596)	(7.674;7.281;7.129)

**Table A20:** Triple of simulated prediction interval lengths averaged over the five different covariance structures for the trigonometric model and r=30% missing rates. The triple covers the sample sizes $\left(n_{1};n_{2};n_{3}\right)=\left(100,500,1000\right)$ using a significance level of α=0.05.

Prediction Interval Length for the Trigonometric Model with *r* = 30% Missings			
Imputation Method	$PI_{n,empQ}$	$PI_{n,ResVar}$	$PI_{n,weighted}$
missForest	(7.581;7.557;7.503)	(8.036;7.769;7.676)	(7.703;7.338;7.204)
mice_pmm	(7.577;7.572;7.526)	(8.036;7.787;7.710)	(7.704;7.357;7.237)
mice_norm	(7.578;7.567;7.524)	(8.036;7.784;7.707)	(7.704;7.355;7.236)
mice_rf	(7.597;7.593;7.548)	(8.061;7.809;7.730)	(7.728;7.378;7.256)
gbm	(7.577;7.552;7.495)	(8.035;7.764;7.667)	(7.702;7.334;7.196)
xgboost	(7.595;7.561;7.508)	(8.047;7.774;7.679)	(7.714;7.344;7.207)

## References

[1] Rubin D.B. (2004). Multiple Imputation for Nonresponse in Surveys.

[2] Enders C.K. (2001). The Performance of the Full Information Maximum Likelihood Estimator in Multiple Regression Models with Missing Data. Educ. Psychol. Meas..

[3] Horton N.J., Laird N.M. (1999). Maximum Likelihood Analysis of Generalized Linear models with Missing Covariates. Stat. Methods Med. Res..

[4] Amro L., Pauly M. (2017). Permuting incomplete paired data: A novel exact and asymptotic correct randomization test. J. Stat. Comput. Simul..

[5] Amro L., Konietschke F., Pauly M. (2019). Multiplication-combination tests for incomplete paired data. Stat. Med..

[6] Amro L., Pauly M., Ramosaj B. (2021). Asymptotic-based bootstrap approach for matched pairs with missingness in a single arm. Biom. J..

[7] Greenland S., Finkle W.D. (1995). A Critical Look at Methods for Handling Missing Covariates in Epidemiologic Regression Analyses. Am. J. Epidemiol..

[8] Graham J.W., Hofer S.M., MacKinnon D.P. (1996). Maximizing the Usefulness of Data Obtained with Planned Missing Value Patterns: An Application of Maximum Likelihood Procedures. Multivar. Behav. Res..

[9] Jones M.P. (1996). Indicator and Stratification Methods for Missing Explanatory Variables in Multiple Linear Regression. J. Am. Stat. Assoc..

[10] Chen H.Y. (2004). Nonparametric and Semiparametric Models for Missing Covariates in Parametric Regression. J. Am. Stat. Assoc..

[11] van Buuren S., Boshuizen H.C., Knook D.L. (1999). Multiple Imputation of Missing Blood Pressure Covariates in Survival Analysis. Stat. Med..

[12] Yang X., Belin T.R., Boscardin W.J. (2005). Imputation and Variable Selection in Linear Regression Models with Missing Covariates. Biometrics.

[13] Sterne J.A., White I.R., Carlin J.B., Spratt M., Royston P., Kenward M.G., Wood A.M., Carpenter J.R. (2009). Multiple imputation for missing data in epidemiological and clinical research: Potential and pitfalls. BMJ.

[14] Stekhoven D.J., Bühlmann P. (2012). MissForest—Non-parametric missing value imputation for mixed-type data. Bioinformatics.

[15] Shah A.D., Bartlett J.W., Carpenter J., Nicholas O., Hemingway H. (2014). Comparison of Random Forest and Parametric Imputation Models for Imputing Missing Data using MICE: A CALIBER Study. Am. J. Epidemiol..

[16] Tang F., Ishwaran H. (2017). Random forest missing data algorithms. Stat. Anal. Data Mining Asa Data Sci. J..

[17] Mayer M., Mayer M.M. Package ‘missRanger’ 2018. https://cran.r-project.org/web/packages/missRanger/index.html.

[18] Chen J., Shao J. (2000). Nearest Neighbor Imputation for Survey Data. J. Off. Stat..

[19] Xu D., Daniels M.J., Winterstein A.G. (2016). Sequential BART for imputation of missing covariates. Biostatistics.

[20] Dobler D., Friedrich S., Pauly M. (2017). Nonparametric MANOVA in Mann-Whitney effects. arXiv.

[21] Ramosaj B., Pauly M. (2019). Predicting missing values: A comparative study on non-parametric approaches for imputation. Comput. Stat..

[22] Zhang X., Yan C., Gao C., Malin B., Chen Y. XGBoost Imputation for Time Series Data. Proceedings of the 2019 IEEE International Conference on Healthcare Informatics (ICHI).

[23] Zhang A., Song S., Sun Y., Wang J. Learning individual models for imputation. Proceedings of the 2019 IEEE 35th International Conference on Data Engineering (ICDE).

[24] Khayati M., Lerner A., Tymchenko Z., Cudré-Mauroux P. (2020). Mind the gap: An experimental evaluation of imputation of missing values techniques in time series. Proc. Vldb Endow..

[25] Bansal P., Deshpande P., Sarawagi S. (2021). Missing value imputation on multidimensional time series. arXiv.

[26] Thurow M., Dumpert F., Ramosaj B., Pauly M. (2021). Goodness (of fit) of Imputation Accuracy: The GoodImpact Analysis. arXiv.

[27] Ramosaj B., Amro L., Pauly M. (2020). A cautionary tale on using imputation methods for inference in matched-pairs design. Bioinformatics.

[28] Meinshausen N. (2006). Quantile Regression Forests. J. Mach. Learn. Res..

[29] Zhang H., Zimmerman J., Nettleton D., Nordman D.J. (2019). Random Forest Prediction Intervals. Am. Stat..

[30] Ramosaj B. (2021). Interpretable Machines: Constructing Valid Prediction Intervals with Random Forests. arXiv.

[31] Ramosaj B., Pauly M. (2019). Consistent estimation of residual variance with random forest Out-Of-Bag errors. Stat. Probab. Lett..

[32] Friedman J.H. (2002). Stochastic Gradient Boosting. Comput. Stat. Data Anal..

[33] Chen T., Guestrin C. Xgboost: A scalable tree boosting system. Proceedings of the 22nd Acm Sigkdd International Conference on Knowledge Discovery and Data Mining.

[34] Chen T., He T., Benesty M., Khotilovich V., Tang Y., Cho H., Chen K. Xgboost: Extreme gradient boosting. R Package Version 0.4-2.

[35] Friedman J.H. (2017). The Elements of Statistical Learning: Data Mining, Inference, and Prediction.

[36] Meng X.L. (1994). Multiple-imputation Inferences with Uncongenial Sources of Input. Stat. Sci..

[37] Fay R.E. (1992). When Are Inferences from Multiple Imputation Valid?.

[38] van Buuren S., Groothuis-Oudshoorn K. (2011). mice: Multivariate Imputation by Chained Equations in R. J. Stat. Softw..

[39] van Buuren S. (2018). Flexible Imputation of Missing Data.

[40] Doove L.L., van Buuren S., Dusseldorp E. (2014). Recursive partitioning for missing data imputation in the presence of interaction effects. Comput. Stat. Data Anal..

[41] Rubin D.B. (1996). Multiple imputation after 18+ years. J. Am. Stat. Assoc..

[42] Ramosaj B. (2020). Analyzing Consistency and Statistical Inference in Random Forest Models. Ph.D. Thesis.

[43] Kuhn M. (2015). A Short Introduction to the caret Package. Found. Stat. Comput..

[44] Ramosaj B., Tulowietzki J., Pauly M. (2021). On the Relation between Prediction and Imputation Accuracy under Missing Covariates. arXiv.

